# Primary adenoid cystic carcinoma of the tracheobronchial tree: report of two cases

**DOI:** 10.11604/pamj.2019.34.137.14902

**Published:** 2019-11-08

**Authors:** Ahmed Ben Saad, Rania Kadoussi, Manel Njima, Saousen Cheikh Mhamed, Nesrine Fahem, Nouha Ben Abdeljelil, Samah Joobeur, Naceur Rouatbi

**Affiliations:** 1 Pulmonology Department, Fattouma Bourguiba Hospital, Monastir, Tunisia; 2 Department of Pathology, Fattouma Bourguiba Hospital, Monastir, Tunisia

**Keywords:** Adenoid cystic carcinoma, salivary gland cancer, lung cancer, bronchogenic carcinoma

## Abstract

Adenoid cystic carcinoma (ACC) is a rare malignant epithelial tumor that predominantly originates in the salivary glands. Primary ACC of the tracheobronchial tree is extremely rare. We report two new cases of central airways primary ACC: a 58 year-old male with an ACC of the left main bronchus who underwent a pneumonectomy with node dissection, and a 52 year-old female with proximal tracheal ACC presenting as asthma treated by surgical resection and a postoperative radiotherapy. Primary ACC of the tracheobronchial tree is often misdiagnosed given the non-specific clinical presentation. An early diagnosis is essential to ensure good outcomes. An interdisciplinary treatment is required based especially on surgery and radiotherapy.

## Introduction

Adenoid cystic carcinoma (ACC) is a malignant tumor that originate mainly from the salivary glands. The pulmonary localization is rare accounting for 0.04 to 0.2% [1]. It is generally located in the proximal tracheobronchial tree. The involvement of the pulmonary parenchyma is exceptional. Primary pulmonary ACC has no specific clinical or imaging features. The diagnosis is confirmed by histopathology. The evolution is generally favorable provided an early diagnosis and a complete surgical resection [2,3].

## Patient and observation

**Figure 1:**
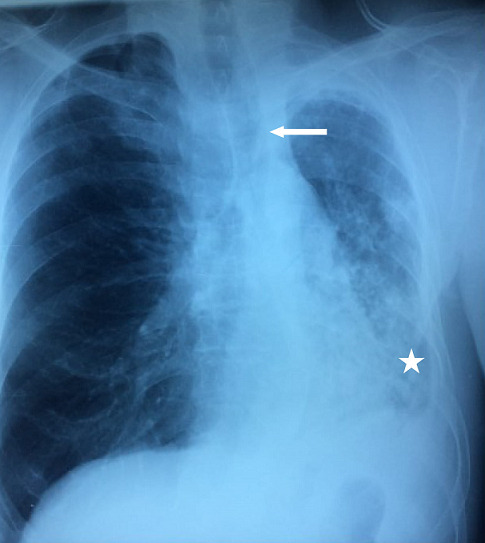
Chest X ray-case 1: persistent left basal consolidation (white star) with a left mediastinal attraction (white arrow)

**Figure 2:**
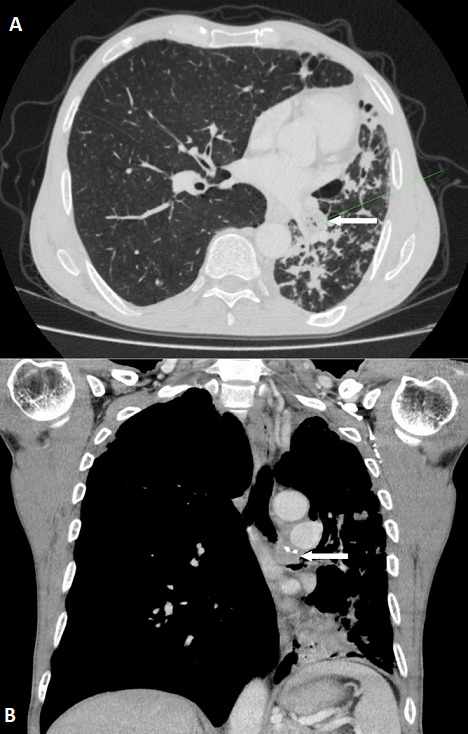
Chest CT scan-case 1: (A) axial CT scan image; (B) coronal CT scan image. Left-sided bronchial-centric mass in the left main bronchus (white arrow) associated with multiple left hilar lymph nodes and carcinomatous lymphangitis in the left inferior lobe

**Figure 3:**
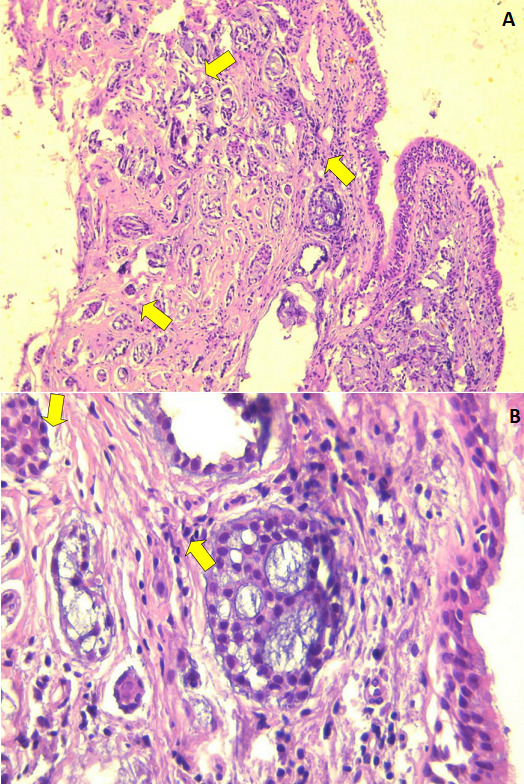
Histopathology: (A) adenoid cystic carcinoma showing cribriform and tubular growth of small hyperchromatic epithelial cells (yellow arrow) (HEx40); (B) tumoral cells are cuboidal with scant cytoplasm and homogenous nuclei (yellow arrow) (HEx400)

**Case 1**: a 58 year- old male with 80 pack-years of cigarettes was hospitalized in our department for the exploration of hemoptysis with cough and sputum, initially diagnosed as pneumonia. There is no history of tuberculosis. Chest auscultation revealed ronchi in left basal region. Complete blood count and blood biochemistry were within normal limits. Sputum was negative for acid fast bacilli or other pathogenic germs. His chest X-ray showed a persistent left basal consolidation (Figure 1). Thoracic CT scan revealed a left-sided bronchialcentric mass in the left main bronchus associated with multiple left hilar lymph nodes and carcinomatous lymphangitis in the left inferior lobe (Figure 2). Cerebral and abdomino-pelvic CT scan did not reveal metastatic lesions. Bronchial fibroscopy showed an endobronchial polypoidal growth in the left main bronchus that entirely occlude the lumen. The histopathological examination of bronchial biopsy showed evidence of adenoid cystic carcinoma (Figure 3). The patient underwent a left pneumonectomy with node dissection. The surgical margins were negative with no node dissemination. A Chest CT performed seven months following surgery didn’t show tumor recurrence. The patient is regularly seen at our consultation. He reports an improvement of his symptoms after the surgery.

**Figure 4:**
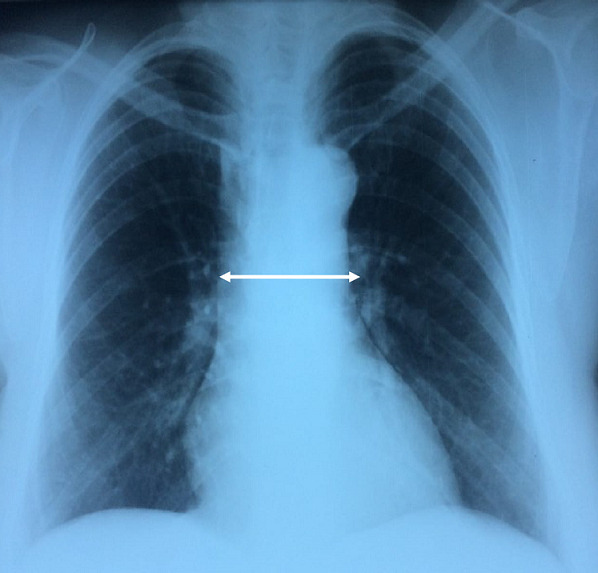
Chest X ray - case 2: mediastinal widening (white arrow)

**Figure 5:**
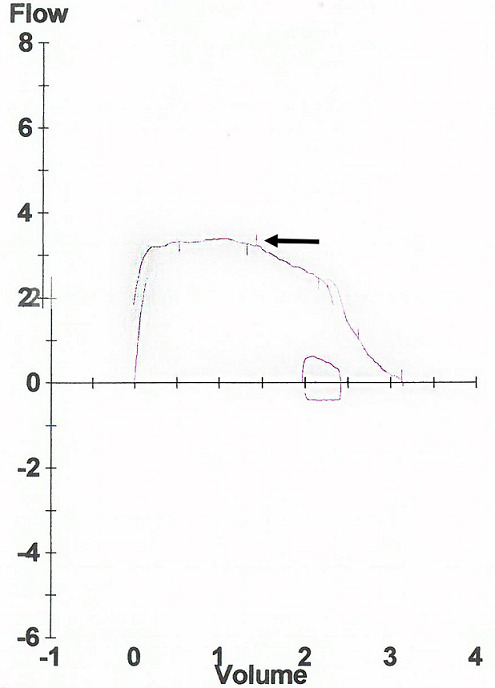
Spirometry: flow volume loop: flattened inspiratory curve (black arrow)

**Figure 6:**
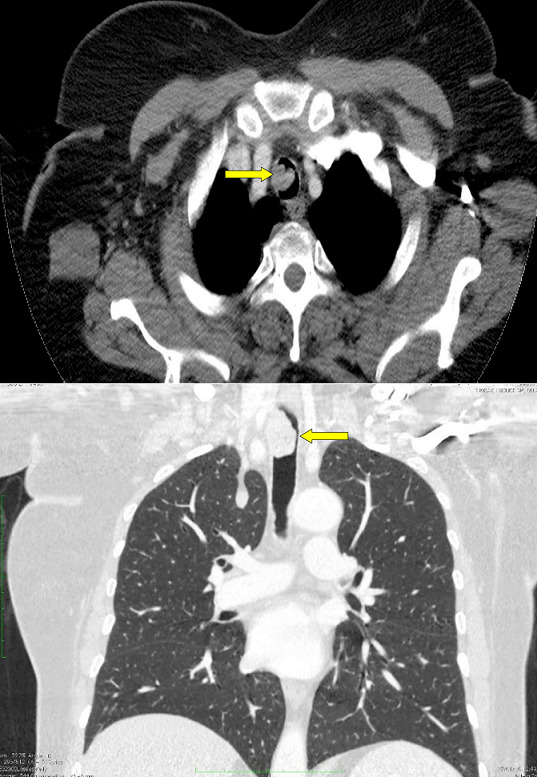
Chest CT scan - case 2: (A) axial CT scan image; (B) coronal CT scan image. Proximal tracheal solid mass (yellow arrow)

**Case 2**: we report the case of a 52 year-old female with insulin-dependent diabetes who was followed as outpatient during four years for cough, wheezing and dyspnea with progressive worsening. The diagnosis of non-allergic asthma was initially suspected and the patient was treated with a long-acting β2 agonist treatment in association with an inhaled corticosteroid. Despite the regular use of an optimal treatment, her symptoms were worsening. Chest X ray showed a mediastinal widening (Figure 4). Spirometry showed a flattened inspiratory curve (Figure 5). The thoracic CT scan revealed a proximal tracheal solid mass (Figure 6). So a Surgical resection of fours tracheal rings was performed. The histopathological analysis concluded to the diagnosis of adenoid cystic carcinoma with positive surgical margins. The patient received postoperative radiotherapy. She had a postoperative follow up of six months with a clear improvement in dyspnea.

## Discussion

Adenoid cystic carcinomas (or cylindroma) are malignant epithelial tumors that have a slow development. It occurs mainly in the salivary glands, but can also affect other sites such as lungs, breasts, skin and uterine cervix [1]. It represents 20 to 35% of tracheal tumors [2]. Primary adenoid cystic carcinoma in the lungs mostly affects the main airway like the trachea or main bronchi. Pulmonary parenchyma involvement is extremely rare [3,4]. It affects both sexes similarly and especially young one aged between 4 th and 5 th decades [1]. Our two patients belong to this interval. There is no evidence of association with tobacco smoking. Clinical manifestations are not specific, dominated mainly by hemoptysis and dyspnea as it was for our patients [5]. Our second case illustrate the example of ACC presenting as asthma. In some cases, the tumor can be asymptomatic resulting in a delayed diagnosis. The histopathological diagnosis is based mainly on the bronchoscopy since the proximal endoluminal growth of the tumor, allowing the realization of a biopsy or a bronchoscope-guided fine-needle aspiration [6]. Hematoxylin-eosin staining remains the principal method used for the diagnosis of ACC.

Immunohistochemistry can be useful in some case. Indeed there is three types of growth to be distinguished on histology: cribriform, tubular and solid. There is a correlation between the evolution of ACC and the histological models. The solid pattern is the most aggressive compared to cribriform [7] .For our patients, the first case was a tubular and cribriform ACC whereas it was of solid type for the second case. The treatment is essentially based on surgical resection for early stage. Postoperative radiotherapy is indicated in the case of positive surgical margins and for non-operable patients [8]. Indeed, ACC is characterized by a possible submucosally and perineurally spread. The place of chemotherapy remains a controversial subject. There is some cases described in the literature that had a response to chemotherapy especially in metastatic cases. The prognosis of ACC depends on histological growth patterns, stage and margins of surgical resection. ACC has a good prognosis with a low metastatic risk especially in patients undergoing radical surgical treatment with clear margins [8]. Otherwise, recurrence or metastases may occur [9,10]. Thus, a prolonged period of follow up is needed.

## Conclusion

ACC is a tumor of good prognosis provided an early diagnosis and rapid management based essentially on surgical resection associated in some cases with radiotherapy. It has no specific clinical presentation leading often to a late diagnosis. More studies are needed to determine the contribution of chemotherapy especially in metastatic tumors.

## Competing interests

The authors declare no competing interests.
